# Integration of AI and ML in regenerative braking for electric vehicles: a review

**DOI:** 10.3389/frai.2025.1626804

**Published:** 2025-09-25

**Authors:** Zacharia Prakash

**Affiliations:** Department of Electrical Engineering, National Institute of Technology Calicut, Kozhikode, Kerala, India

**Keywords:** artificial intelligence, machine learning, energy recovery, fuzzy logic, neural networks, regenerative breaking, reinforcement learning, electric vehicles

## Abstract

Electric vehicle technology has grown rapidly in recent years due to battery advancements, environmental concerns and supportive policies. Regenerative braking systems play a critical role in improving energy efficiency by converting kinetic energy into electrical energy, thereby extending battery life and vehicle range. However, conventional regenerative braking faces challenges in energy recovery, comfort, and adaptability. Optimizing energy recovery ensures prolonged battery life by preventing overcharging or undercharging, making EVs more sustainable and cost-effective. This review paper explores the integration of Artificial Intelligence and machine learning techniques in regenerative braking systems to overcome these challenges. This study examines AI techniques such as regression models, neural networks, deep reinforcement learning, fuzzy logic, genetic algorithm and swarm intelligence based techniques for regenerative braking. The study also compares AI-based strategies with traditional braking methods. Unlike previous studies, which focus on individual AI techniques, this paper provides a comparative analysis of multiple AI approaches, assessing their impact on braking performance and energy recovery, and propose a hybrid AI framework. This paper covers challenges in real-time implementation, road adaptability, and vehicle control integration. This paper also discusses future research that optimize braking performance like V2X communication, edge computing, and explainable AI etc.

## 1 Introduction

**Figure 1:**
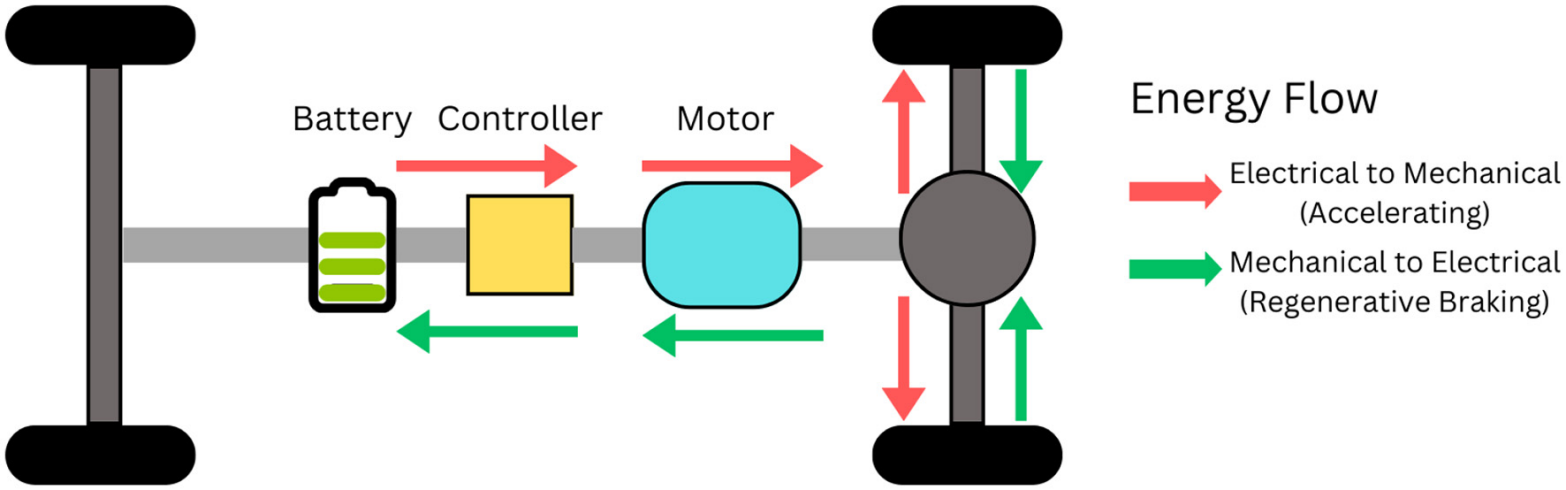
A schematic representation of a regenerative braking system illustrating the bidirectional energy flow between the vehicle's wheels and the battery during deceleration and acceleration phases.

Electric vehicle (EV) adoption has surged due to breakthroughs in battery technology, stringent government policies, and increasing environmental concerns. The International Energy Agency forecasts EVs will capture a 35% global market share by 2030 (Zaino et al., 2024). Among the key drivers of EV efficiency is regenerative braking, a process where kinetic energy from deceleration is converted and stored as electrical energy in the battery rather than lost as heat. As illustrated in Figure 1, this bidirectional energy flow allows the motor to act as a generator during braking, dramatically enhancing energy recovery, extending driving range, and potentially prolonging battery life. Recent advances in artificial intelligence (AI) and machine learning (ML) are now poised to revolutionize regenerative braking systems (RBS) by enabling smarter, adaptive control strategies that optimize energy capture while maintaining safety and drivability. AI/ML techniques can dynamically adjust braking force, learn from varying road and driving conditions, and predict optimal energy recovery patterns in real time, addressing limitations of traditional rule-based methods. This integration marks a transformative step toward maximizing

EV efficiency and sustainability, setting the stage for the next generation of intelligent regenerative braking technologies (Mitropoulos-Rundus et al., 2021).

In this paper, a conventional regenerative braking system refers to one with a fixed brake force distribution between the front and rear axles, unable to adapt to changes in road conditions, vehicle load, or tire-road adhesion. This static approach often results in lower energy recovery and reduced stability in varied driving scenarios. When braking, the electric motor acts as a generator, feeding energy back into the battery (Chandak and Bhole, 2017). Unlike traditional service braking, regenerative braking initiates deceleration as soon as the accelerator is released (Mitropoulos-Rundus et al., 2021). Control strategies such as fuzzy logic and PID improve braking force distribution, ensuring smooth transitions and better energy recovery (Nian et al., 2014). ML methods further optimize recovery by considering factors like battery SOC, braking demand, and real-time conditions (Prasanth et al., 2023). Beyond energy capture, AI models have also shown potential for fault diagnosis (Sankavaram et al., 2014) and optimizing braking performance under varying traffic conditions (Zhang et al., 2020).

In conventional regenerative braking systems, a substantial portion of braking energy is lost due to inefficient conversion, with less than 50% recovered in urban driving and even lower efficiency in high-speed or emergency braking scenarios (Yin et al., 2023a). These systems also lack adaptability, operating with fixed front–rear braking force ratios regardless of road conditions, load, or adhesion, leading to suboptimal energy recovery (Yin et al., 2023b). Moreover, regenerative braking alone cannot supply the force needed for sudden or rapid deceleration, necessitating mechanical braking and further reducing recovery efficiency (Hwang et al., 2023). ML models address these limitations by predicting braking force from real-time inputs such as SOC, speed, road conditions, and driving patterns to optimize recovery. AI-based control dynamically adjusts force distribution, enhancing stability, comfort, and efficiency under varying and emergency conditions. Key approaches include supervised learning (e.g., regression models, neural networks) for force prediction, reinforcement learning (e.g., Q-learning, deep Q-networks) for adaptive braking, and fuzzy logic for smooth transitions. Hybrid AI models integrate these methods to balance energy recovery with vehicle stability.

**Figure 2:**
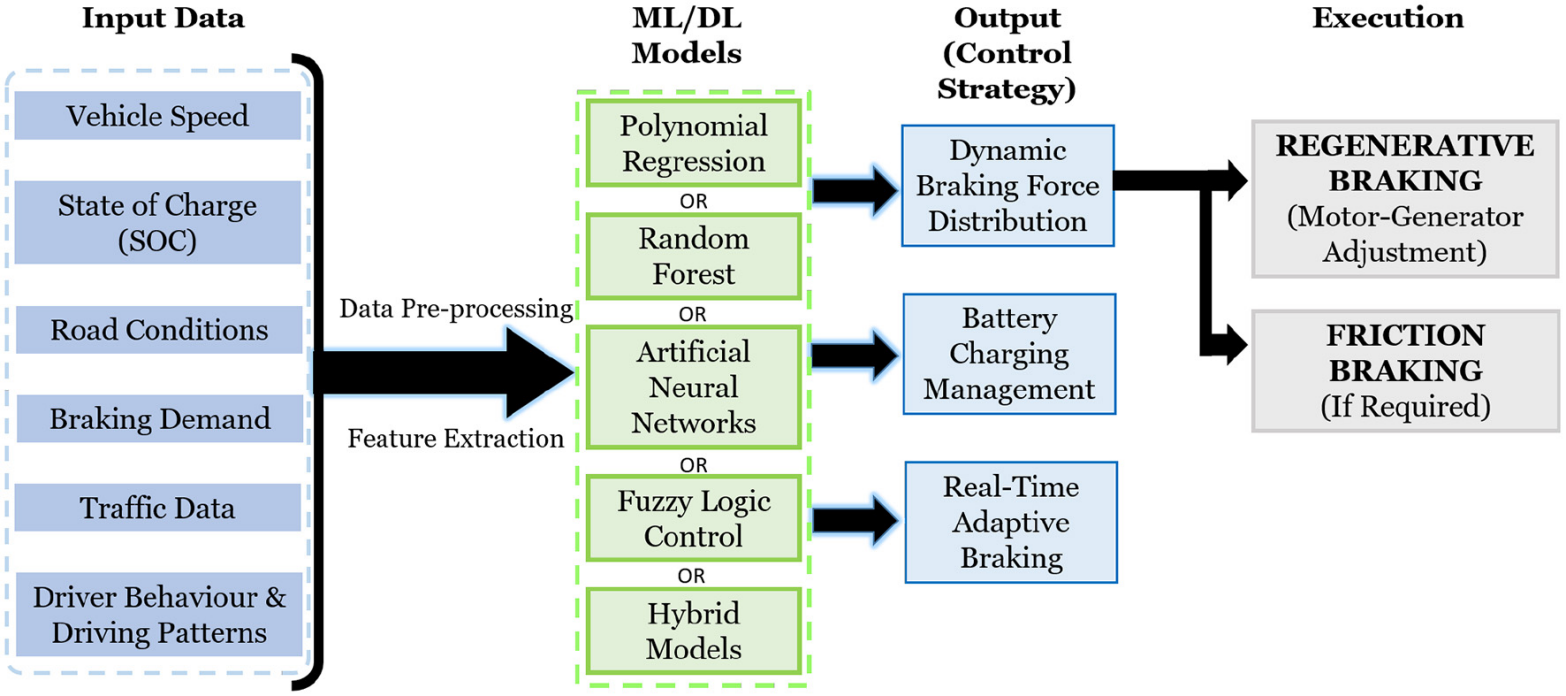
A general architecture of an AI/ML-based regenerative braking system illustrating the flow of input variables into machine learning models for deriving optimized braking control strategies.

Existing research demonstrate diverse AI/ML applications in optimizing regenerative braking systems. Supervised learning models, including Polynomial Regression and Random Forest, predict energy generation and optimize power distribution between storage units, achieving up to 59% improvement over traditional methods (Prasanth et al., 2023). Fuzzy Logic Controllers dynamically adjust braking force based on variables like vehicle speed, battery SOC, and braking intensity (Xu et al., 2011). Reinforcement Learning, using state-action-reward frameworks and Deep Q-Networks, enhances braking stability and energy recovery in simulations (Chae et al., 2017). Neural Networks, such as Bidirectional LSTM and ANN-IWHO, optimize torque allocation and driving comfort across different road conditions (Chae et al., 2017). Genetic Algorithms improve driving patterns and braking settings, with GA-based eco-driving extending vehicle range and hybrid GA-fuzzy systems reducing heat generation (Gautam et al., 2021; Arunprasad et al., 2023). These techniques collectively demonstrate AI/ML's capability to balance energy recapture, vehicle stability, and battery longevity. However, most studies focus on individual methods without comparative analyses under standardized conditions. Moreover, experimental validation is limited, heavily relying on simulations, which raises concerns about real-world applicability amid traffic variability, road conditions, and computational limits (Zhang et al., 2020). Few works address regenerative braking's impact on battery health (Chidambaram et al., 2023). Integrating AI/ML into existing braking systems remains challenging due to nonlinear dynamics and safety demands. Future work should emphasize comparative studies and real-world testing to bridge these gaps. Figure 2 depicts the overall AI/ML regenerative braking architecture, showing how inputs like battery SOC, vehicle speed, and braking demand feed into ML models to optimize control strategies, highlighting the importance of integrated AI solutions.

The primary objective of this review is to present a comprehensive analysis of AI/ML integration in regenerative braking systems (RBS) for electric vehicles. It examines major AI-driven approaches such as fuzzy logic controllers, neural networks, reinforcement learning, and genetic algorithms and evaluates their contributions to energy recovery, braking efficiency, stability, and adaptability. Performance comparisons are made under standardized conditions, including energy recapture rates and real-world applicability. The review also identifies key gaps, notably the scarcity of experimental validation, limited cross-technique comparative studies, and challenges in integrating AI into practical braking systems. By synthesizing existing research, this work highlights AI/ML's potential to enhance efficiency, safety, and overall vehicle performance while outlining future directions for advancing regenerative braking technologies.

This review examines AI/ML techniques applied to regenerative braking in electric vehicles, focusing on energy recovery efficiency, braking comfort, and vehicle adaptability as primary evaluation metrics (Wager et al., 2018). It compares approaches including fuzzy logic, neural networks, genetic algorithms, and reinforcement learning based on energy recovery, braking force distribution, stability, and comfort. The assessment draws majorily on MATLAB/Simulink simulations but limited real-world applications (Saiteja et al., 2022), addressing practical challenges such as real-time adaptability, computational demands, and integration with existing vehicle systems. While highlighting AI-driven advantages like dynamic optimization and increased energy savings, the review acknowledges limitations involving data dependency and computational complexity. It excludes hardware specifics, economic factors, regulatory policies, and detailed mechanical aspects, and does not provide an in-depth discussion of fault diagnosis (Sankavaram et al., 2014) due to limited comprehensive research. This focused analysis is confined to software-based control strategies, emphasizing AI/ML's role in optimizing regenerative braking performance.

The review methodology follows systematic literature review practices tailored for engineering and AI, covering publications from 2010 to 2025 via databases like IEEE Xplore, ScienceDirect, SpringerLink, Web of Science, and Google Scholar. Relevant studies involving regenerative braking, EVs, AI, machine learning, and specific techniques were screened through a two-stage process, focusing on peer-reviewed, English-language works. Extracted data on methods, metrics, and findings were synthesized to reveal comparative trends, real-world constraints, and research gaps, adhering to standards of transparency and reproducibility.

The structure of this review is organized as follows: Section 1 provides an introduction to the study. Section 2 covers the basics of regenerative braking, including how it works, the different types, and the main challenges involved. Section 3 looks at different AI and ML methods and explains how each one helps improve the regenerative braking system. Section 4 compares these strategies with traditional braking methods, focusing on how well they recover energy, provide comfort, and adapt to different driving conditions. Section 5 proposes a hybrid AI framework that integrates the strengths of multiple techniques. Section 6 discusses the major implementation challenges of AI/ML models in braking systems and highlights future research directions. Finally, Section 7 presents the discussion and concluding remarks.

## 2 Fundamentals of regenerative braking

### 2.1 Working principles

In electric vehicles, regenerative braking converts kinetic energy into electrical energy during deceleration by switching the wheel motor into generator mode. BLDC motors are often used for their efficiency and dual motor/generator capability (Nian et al., 2014). Main system components include wheel motors, bidirectional power electronics (e.g., H-bridge inverters with MOSFET/IGBT switches), and energy storage units like lithium-ion batteries or supercapacitors. Braking triggers the motor to act as a generator, inducing back EMF that charges the battery via the bidirectional converter. Dual-motor all-wheel-drive EVs further enhance energy recovery compared to single-motor setups (Heydari et al., 2020).

### 2.2 Types of braking

Braking systems in electric vehicles are categorized into three: mechanical, regenerative, and hybrid. Mechanical braking relies on friction, turning kinetic energy into heat and causing wear, but provides crucial safety. Regenerative braking slows the vehicle by using the motor as a generator to recover energy, though its effectiveness is limited when the battery is fully charged or at low speeds. Hybrid systems integrate both methods, ensuring reliable deceleration across all conditions by compensating for the limitations of regenerative braking, especially during low speeds or full battery scenarios (Da and Bo, 2015). In emergency braking, the system defaults fully to friction braking for maximum safety. Thus, the best systems balance efficient energy recovery with uncompromised safety.

### 2.3 Challenges

Regenerative braking systems must balance energy recovery with braking stability and safety. At low speeds, conversion efficiency drops significantly. Emergency stops require deceleration forces that exceed the capacity of regenerative systems. Frequent charge and discharge cycles also accelerate battery degradation. High current charging, especially at high temperatures or elevated state of charge (SOC), increases the risk of lithium plating and capacity loss. Over time, this cycling reduces battery storage ability and overall vehicle performance. Although regenerative braking can extend lithium-ion battery life by reducing deep discharges (Chidambaram et al., 2023), frequent high SOC charging especially in warm conditions can speed-up degradation. While regenerative braking mainly accelerates cycling aging, constant high SOC and elevated temperatures also drive calendar aging during storage (Keil and Jossen, 2017). To prevent damage, battery management systems must carefully regulate charging rates, SOC, and temperature to optimize both energy recovery and long-term battery health. Transition lag between mechanical and regenerative braking further complicates stability during rapid stops.

## 3 AI/ML-based approaches for regenerative braking

### 3.1 Regression models

Polynomial Regression (PR) and Random Forest Regression (RFR) are used to optimize regenerative braking energy in electric vehicles. PR models braking force as a nonlinear function of inputs such as brake demand and battery SOC, using gradient descent for optimization and incorporating principal component analysis (PCA) to reduce dimensionality. RFR, which combines predictions from multiple decision trees trained on random data subsets, identifies brake demand as the important factor for accurate braking force prediction. RFR achieved better accuracy with a lower root mean square error (RMSE) of 5.16e-04 compared to PR's 0.042588 and handled nonlinear patterns more effectively. However, PR produced higher regenerative force in most driving conditions. For example, 16,000 W vs. 15,770 W in FTP cycles (Prasanth et al., 2023). Both models underperform relative to real-time optimization (RTO) methods but offer useful compromises: PR is faster and suitable for real-time use, whereas RFR manages complex nonlinearities better but with higher computational delay. A major limitation of PR is its depends on fixed polynomial terms, which can result in underfitting (Kim et al., 2021). Overall, both techniques improved energy recovery by approximately 59% compared to traditional fuzzy logic and neural network approaches (Prasanth et al., 2023).

### 3.2 Neural networks

**Figure 3:**
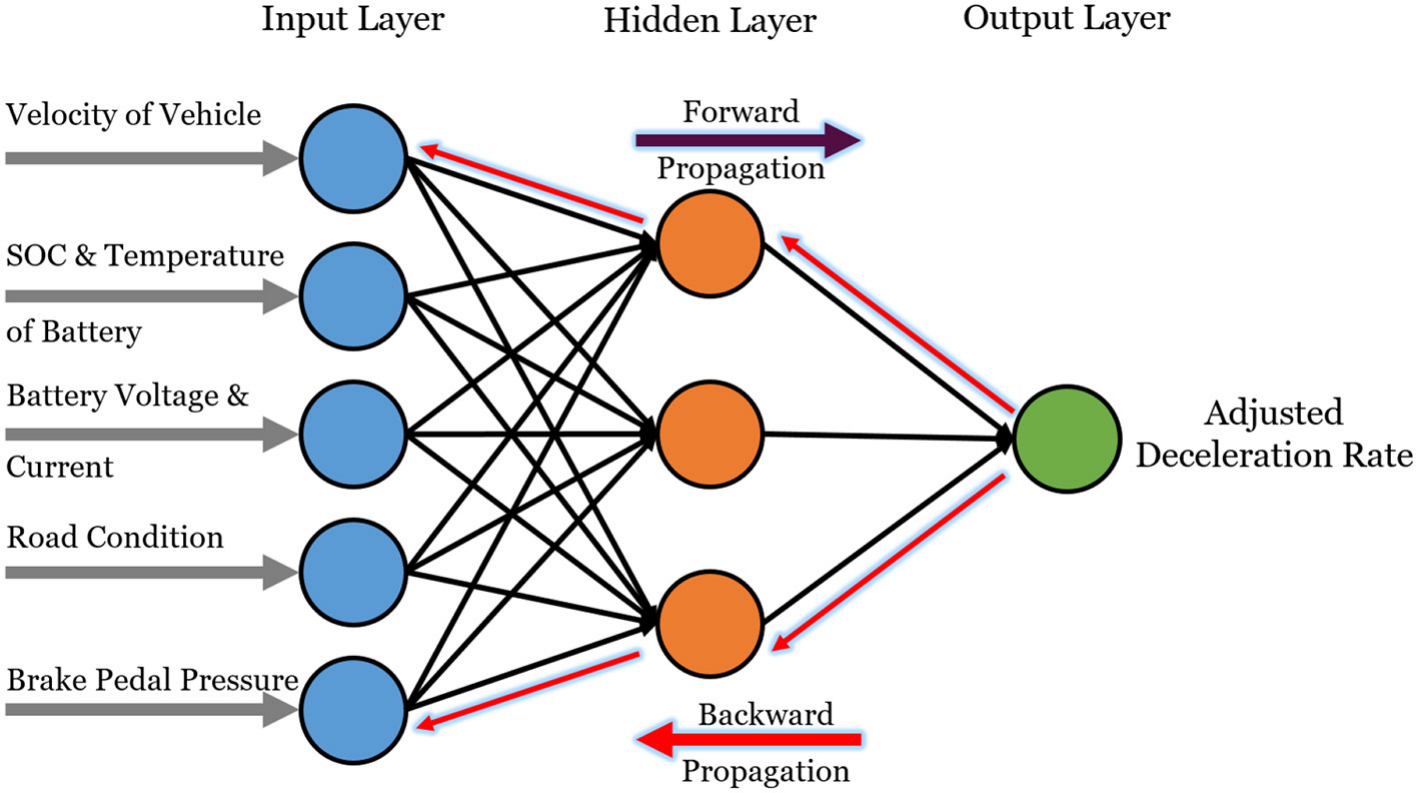
A neural network architecture for braking force optimization utilizing backpropagation for adaptive learning and control refinement.

Artificial neural networks (ANNs) predicts braking force by analyzing inputs like battery SOC, speed, and brake demand. ANNs minimizes prediction errors by continuous weight updation which enables the network to learn input-output relationships. Unlike traditional experimental or rule-based approaches, the ANN model offers faster, more flexible predictions and enables real-time control system development without physical trials (Rezk and Abuzied, 2023). The neural network (NN) architecture for braking force optimization is illustrated in Figure 3. The studies such as Velu and Chellammal (2023) introduce a novel Improved Wild Horse Optimization Algorithm (IWHO) to optimize ANN weights, achieving good accuracy. This approach maximizes energy recovery by 15% while ensuring vehicle stability through optimal brake force distribution. Models like Long Short-Term Memory (LSTM) and Nonlinear Autoregressive Exogenous (NARX) can predict how much energy can be recovered during braking (Ziadia et al., 2023). These models consider driver-specific regeneration limits and road topology into optimization, achieving 16%–39.6% energy recovery improvements across various driver cycles. It is expected that this adaptive approach can reduce computational load while outperforming traditional methods in downhill scenarios.

In Neural Inverse Optimal Control (NIOC), a special type of neural network called a Recurrent High-Order Neural Network (RHONN) is trained using an Extended Kalman Filter (EKF) (Ruz-Hernandez et al., 2022). This training helps the network understand how voltage and current behave in a buck-boost converter. This approach enables accurate tracking of time-varying references and optimizes energy recovery during braking without solving complex equations. It achieves high tracking accuracy and improves SOC retention by 20.41%. Neural network models like LSTM and Transformer help estimate energy use during braking by learning from how the vehicle is driven and the road conditions (Zhang et al., 2024). This helps in dynamically estimating regenerative braking efficiency, which traditional models often oversimplify with static coefficients while achieving nearly 15% lower errors.

However, ANN performance relies heavily on training data quality. It requires large datasets and face computational complexity and makes real-time deployment difficult. Additionally, the “black-box” nature of NN reduces interpretability compared to rule-based models (Zhang et al., 2024). Also for Optimization Algorithms like IWHO, computational complexity would make real-time implementation difficult. Although this method performs better than traditional fuzzy logic and many ANN approaches, it shows poor real-time optimization in dynamic energy recovery.

### 3.3 Deep reinforcement learning

Deep Reinforcement Learning (DRL) is a method where an agent learns to make decisions by interacting with an environment. It uses deep neural networks to understand complex patterns and improve actions over time. The agent gets rewards for good actions and learns to avoid bad ones. Over many tries, it figures out the best way to reach a goal. DRL is being used in areas such as modern autonomous vehicles and related energy systems. For instance, Min et al. (2019) proposes a multi-level deceleration algorithm that blends driver modeling and optimization-based techniques to identify optimal deceleration trajectories. In another study (Kim et al., 2021), the regenerative braking process is modeled using parameterized polynomial deceleration and utilizes dynamic programming to maximize energy recapture during braking events. By integrating external factors such as traffic signals and road topography, the system dynamically adjusts deceleration commands, yielding at least a 16% increase in energy recovery and a 3%-10% reduction in trip time compared to human drivers, while maintaining a 3-meter safety margin in 95% of test cases. Enabling V2I (vehicle-to-infrastructure) communication can help in recovering more energy when braking near traffic lights (Zhang et al., 2020). Here the braking process is set up as a decision-making problem using RL, where the vehicle's distance and speed be the “state” and different ways of slowing down are the “actions.” The algorithm tries different actions, learns from the results, in order to maximize the rewards- balances energy recovery and smooth driving. With the available real-time traffic light data, two RL methods were tested in the studies, Q-Learning and Deep Q-Network(DQN). The Q-learning method recovered 45.08% more energy than simple uniform braking and DQN recovered 2.24% more energy and demonstrated smoother braking.

To resolve the inherent trade-offs between different system objectives (energy recovery, comfort, and safety), DRL algorithms commonly use a custom reward function to that quantifies these often conflicting objectives. One such representative reward function *r*_*t*_ at time step *t* is formulated as:


$$r_{t}=\lambda_{1}\cdot \frac{E_{\text{regen}}\left(t\right)}{E_{\max}}-\lambda_{2}\cdot {\left(a_{\text{desired}}-a_{t}\right)}^{2}-\lambda_{3}\cdot I\left(\text{violation}\right)$$

Here *E*_regen_(*t*) denotes the energy recovered during braking, normalized by the maximum possible recoverable energy *E*_max_. The term ${\left(a_{\text{desired}}-a_{t}\right)}^{2}$ penalizes deviations from the desired deceleration *a*_desired_, promoting smooth and comfortable braking. The indicator function *I*(violation) imposes penalties for unsafe conditions, such as excessive wheel slip or insufficient safety margins. The weighting factors λ_1_, λ_2_, and λ_3_ balance the trade-offs between maximizing energy efficiency, maintaining braking comfort, and ensuring safety.

This reward structure enables the DRL agent to dynamically adjust braking strategies, optimizing energy recapture while preserving vehicle stability and passenger comfort. It clearly expresses the key algorithmic foundation in many DRL-based regenerative braking systems.

Recent advancements demonstrate the diverse potential of DRL in this field. For example, the Deterministic Policy Gradient (DPG) approach has been applied to develop adaptive regenerative braking strategies for pure electric vehicles, leading to 57.69% faster convergence and nearly 2% lower energy consumption compared to traditional control, as well as extended battery life (Xu, 2025). Another framework (Ramesh et al., 2024) combines DRL with modular recurrent neural networks (RNNs) for tasks such as localization, path planning, and power management. The algorithm leverages real-time sensor and historical data, with modular RNNs reducing overall power consumption via time-series memory reuse, ultimately showing improved energy efficiency in highway scenarios. Further, the use of the Twin Delayed Deep Deterministic Policy Gradient (TD3) algorithm enables dynamic allocation of braking torque across motors and hydraulic systems, delivering 35.18% energy recovery under various driving cycles (Peng and He, 2025). In more complex hybrid setups, Fuzzy Q-Learning (FQL) has been used to optimize hydraulic regenerative braking by dynamically adjusting force in real time, also introducing trauma memory to enhance safety in rare collision scenarios (Ning et al., 2023). These models achieved energy recovery up to 9.62% higher than standard fuzzy control and successfully maintained strict safety margins. Both Kim et al. (2021) and Ning et al. (2023) report 100% success in Euro NCAP AEB tests at 60 km/h, along with smooth, efficient deceleration profiles—a notable improvement over conventional rule-based approaches.

### 3.4 Fuzzy logic models

**Figure 4:**
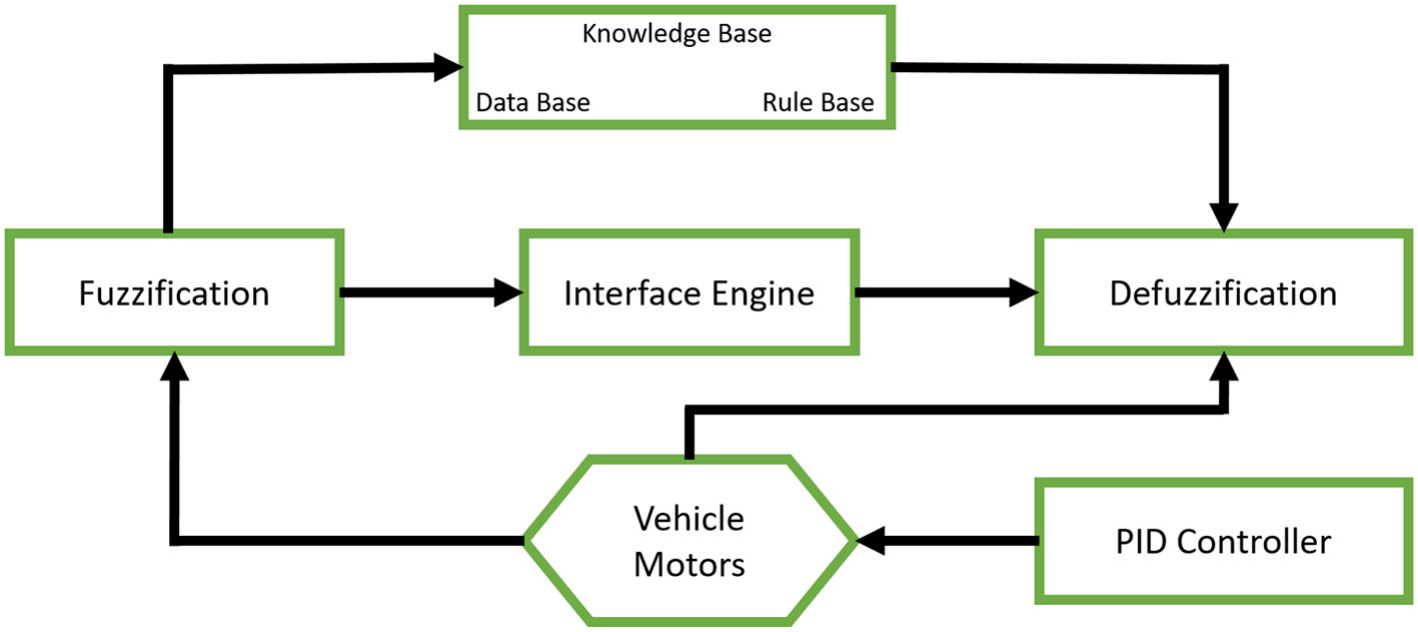
Schematic representation of a fuzzy logic-based PID controller illustrating the integration of fuzzy inference with traditional PID control for enhanced braking force optimization.

Fuzzy logic models work in a way that's similar to how humans make decisions. Membership functions in fuzzy logic is used to define how each input value is mapped to a degree of membership between 0 and 1. They help translate real-world inputs (like battery SOC, temperature, vehicle speed, or brake requirement) into fuzzy values like “low,” “medium,” or “high,” this process is also known as fuzzification. This allows the system to handle uncertainty and make decisions in a way that mimics human reasoning. The result is defuzzified to get a crisp output. Fuzzy logic is one of the most commonly used RBS techniques according to existing literature. Most of the papers use fuzzy logic as a method to improve the existing methods such as PID controllers, reinforcement learning or neural networks. The integration of fuzzy inference systems with conventional PID controllers for braking force optimization is shown schematically in Figure 4.

Advanced fuzzy logic techniques, such as Type-2 Fuzzy Logic (IT2FL) (Wahid et al., 2024), have been used to optimize regenerative braking in electric trikes, utilizing the Nie-Tan method as a type-reducer to streamline computation. This approach improved energy recovery by 3% (2.3 km range extension) over conventional fuzzy systems and delivered 35.84% total energy recovery (91 km increase) and 50 km/kWh efficiency (41.7% improvement) compared to non-regenerative control. Other works (Islam et al., 2023) integrate fuzzy logic with PID controllers, resulting in 8%–25% greater driving range and enhanced stability. In fuzzy models such as Maia et al. (2020) a technique called Batch Fuzzy Q-Learning (BFQL) is used to optimize the regenerative braking factor (RBF). Integrating this reinforcement learning technique eliminates the need for manual expert adjustments to automate the tuning of fuzzy rules. This approach improved energy recovery predictions, reducing errors by up to 22.47% compared to manual methods.

Gradient-based fuzzy logic controllers (Vodovozov et al., 2020) further adapt braking force to changing road conditions, facilitating smooth shifts between normal and anti-lock modes. Simulations report recovery of 22 kJ even under variable tire-road friction. Two-layer fuzzy-PID control strategies (Anh et al., 2024) separate front/rear braking force allocation (using ideal braking curves) from the real-time fuzzy distribution, yielding 13%–30% energy recovery gains. Takagi–Sugeno fuzzy sliding-mode controllers (TSFSMC) (Zhang et al., 2016) ensure above 91% efficiency without additional sensors by ensuring precise voltage regulation and fast responsiveness in dynamic operating conditions.

A two-layer fuzzy logic control (FLC) system (Yin et al., 2023a) employs 64 fuzzy rules and centroid defuzzification to dynamically determine braking ratios, with integrated PID controllers ensuring stable nonlinear dynamics and achieving up to 40% simulated energy savings. Hybrid approaches using PID controllers combined with Adaptive Neuro-Fuzzy Inference System (ANFIS) (Akhila and Ratnan, 2016) deliver rapid response and adaptability to changing conditions, resulting in a 50% increase in driving range and superior battery management vs. stand alone PID or FLC. Takagi–Sugeno (T-S) type fuzzy neural networks (FNN) (Li W. et al., 2024) can self-optimize fuzzy rules, enhancing flexibility, accuracy, and driving range by 19.2%. For real-time driving-mode switching, fuzzy logic-based decision systems (Ning et al., 2023) optimize transitions between single-motor and dual-motor modes, using an evaluation layer to minimize unnecessary switching and mechanical wear, while improving overall system stability.

A notable study on hybrid energy storage systems (HESS) combining supercapacitors and batteries (Li W. et al., 2024) demonstrates that a FLC can dynamically split braking energy, prioritizing the supercapacitor during high-energy events to reduce strain on battery. This control approach achieves a 29.1% reduction in battery current and a 46.84% decrease in heat generation, thus extending battery life. Separately, a fuzzy logic-based regenerative braking system using a Sugeno-type controller (Xu et al., 2011) introduces innovations like series braking (allowing independent control of the front and rear axles) and acceleration sensors that align braking distribution with ideal curves—effectively preventing wheel lock and enhancing stability. This system leads to a 25.7% increase in driving range and a 22.2% improvement in energy efficiency compared to vehicles without coordinated fuzzy braking control.

The major drawback of most of the fuzzy logic based RBS is the complexity of tuning membership functions, especially under variable and nonlinear driving conditions. Managing rapid SOC fluctuations while balancing real-time responsiveness with computational efficiency make real-world implementation challenging. In Şen et al. (2024) and Wahid et al. (2024) which has hybrid energy systems, managing the fast interaction between the super-capacitors and battery is also an important concern.

### 3.5 Genetic algorithms

Genetic Algorithms (GAs) operate by evolving a population of candidate solutions through processes such as selection, crossover, and mutation, ultimately converging on optimal or near-optimal solutions. Unlike gradient-based methods, GAs are well-suited for complex, multi-modal problems where derivative information may not be available. In regenerative braking optimization, GAs evaluate and refine braking control settings across generations, prioritizing energy recovery and safety.

For example, a GA-based eco-driving approach for electric vehicles (Gautam et al., 2021) optimized acceleration profiles and cruising speeds, successfully minimizing energy consumption. This method effectively avoided local optima and managed computational complexity, outperforming a Stochastic Hill Climber (SHC) method by achieving greater energy savings. However, it faced challenges with the encoding of control variables as 14–32 bit chromosomes and with balancing search space efficiency. In another context, a GA-driven regenerative braking system for railways (Che et al., 2022) was developed to optimize power distribution through railway power networks and energy converters. In hardware-in-loop tests, this strategy realized 93.3% utilization of regenerative braking energy—significantly surpassing the SHC approach and reduced three-phase current imbalance by 98.6%, a critical improvement for railway systems.

Some studies propose hybrid optimization schemes combining Genetic Algorithms (GA) with fuzzy logic for enhanced regenerative braking. For instance, Bostanian et al. (2013) details a GA-tuned FLC strategy that optimizes the split of braking torque between mechanical and regenerative systems during deceleration, resulting in a 26% reduction in both energy costs and emissions compared to standard controllers. A key challenge was integrating 16 control settings into the GA, while ensuring vehicle performance constraints were still satisfied. For another approach, the hybrid neuro-fuzzy-genetic algorithm (NFGA) (Arunprasad et al., 2023), uses neural networks for system modeling, fuzzy logic for decision processes, and GA for optimal parameter tuning. This comprehensive framework achieved a 40% reduction in control deviation and substantially improved trajectory tracking accuracy, demonstrating the value of combining soft computing and evolutionary techniques in RBS control.

### 3.6 Swarm intelligence-based algorithms

Swarm intelligence based approaches for regenerative braking uses biologically inspired collective problem-solving to optimize energy recovery. In Particle Swarm Optimization (PSO), each “particle” evaluates candidate solutions and moves according to both its own historical best and the swarm's collective best, iteratively converging on optimal braking force allocation. Ant Colony Optimization (ACO) uses artificial “ants” to explore potential strategies, with solution quality guiding future searches via virtual pheromone trails. A recent study (Chai et al., 2022) applied PSO to tune a PI controller for regenerative braking in electric vehicles using super-capacitors. This optimization produced a 6% increase in travel distance, a 14.57% rise in average speed, a 3.69% improvement in maximum speed, and lower speed tracking errors. However, the increased energy recovery demanded careful management of super-capacitor SOC, which dropped by 1.58% due to higher usage. The PSO-selected controller parameters (*K*_*p*_ and *K*_*i*_) also enhanced BLDC motor performance during braking, maintaining voltage stability (with a constant 540V initial supercapacitor voltage) throughout deceleration events.

**Figure 5:**
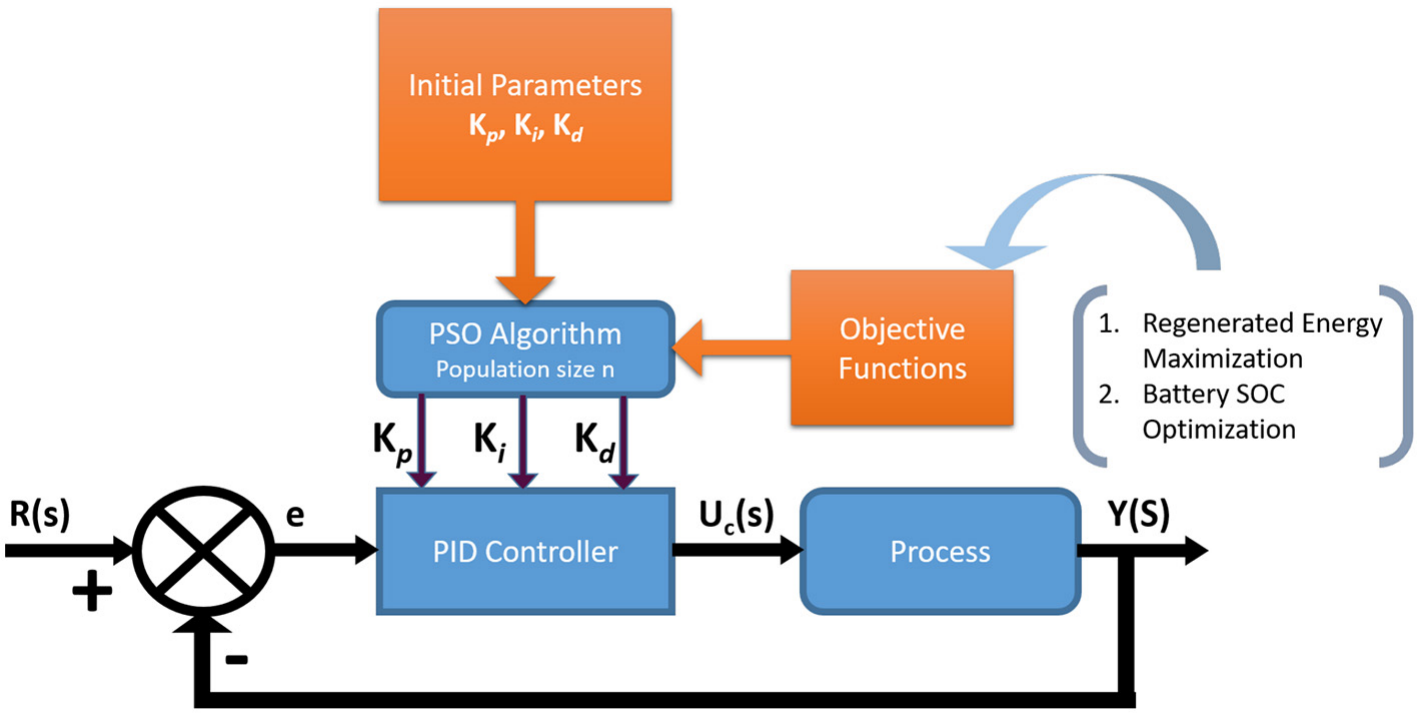
Schematic representation of a PSO-optimized PID controller in regenerative braking illustrating the use of particle swarm optimization for tuning PID parameters to enhance energy recovery and braking efficiency.

The paper (Pradhan et al., 2023) proposes a PSO-optimized PID controller for brushless DC (BLDC) motor speed control in electric vehicles. This method aimed at improving regenerative braking efficiency such that it automatically tunes the PID gains (*K*_*p*_, *K*_*i*_, *K*_*d*_) using PSO, resulting in faster settling time, reduced speed tracking errors, and a 3% reduction in SOC loss during braking compared to conventional PID tuning. Figure 5 illustrates the overall architecture of a PSO optimized PID controller, highlighting how the PSO algorithm dynamically adjusts PID parameters in response to real-time driving conditions. Such AI-based adaptive control yields improved energy recovery, quicker response to disturbances, and enhanced robustness over traditional control strategies, as confirmed by recent simulation results.

Another approach employs PSO in combination with fuzzy logic to optimize regenerative braking force distribution in automobiles (Tuan et al., 2020). This method achieved a 10.49%–24.44% reduction in fuel consumption by maximizing both the duration and efficiency of regenerative braking, particularly surpassing conventional fuzzy logic strategies in low-speed scenarios. Further advancements are seen in the use of swarm intelligence-based hybrid strategies that combine PSO and ACO to optimize braking torque distribution in hybrid electric vehicles (Zhang et al., 2022, 2019). Specifically, Zhang et al. (2019) reports a 16.04% increase in energy recovery compared to rule-based methods. Key findings include a 63.51% regenerative energy recovery rate in urban driving cycles (Zhang et al., 2022), outperforming both PSO-only (62.26%) and rule-based (54.24%) approaches. The principal advantage of these hybrid PSO-ACO strategies lies in their ability to avoid local optima, ensuring stable slip ratios (around 0.2) during emergency braking, while simultaneously balancing considerations related to battery aging and safety.

## 4 Comparative analysis of AI-based and conventional regenerative braking strategies

Conventional rule-based methods for regenerative braking rely on predefined logic and fixed thresholds to allocate braking force between regenerative and friction braking systems. These strategies uses static rules based on parameters such as braking intensity, battery SOC and vehicle speed to determine when and how much regenerative braking should be applied. For example, the logic threshold control strategy described in Yin et al. (2023b) divides braking into zones:

- Low-intensity braking uses only regenerative braking,
- Moderate intensity combines regenerative and hydraulic braking,
- High intensity prioritizes hydraulic braking for safety.

While this approach is simple and easy to implement, it lacks adaptability to changing driving conditions and cannot dynamically respond to changing road, driver, or battery scenarios. As a result, rule-based methods often lead to lower energy recovery and reduced system efficiency under complex or frequently changing environments. Similarly, in Da and Bo (2015) rule-based mode switching was found to cause frequent, unnecessary transitions between regenerative and friction braking, reducing efficiency and stability compared to adaptive fuzzy logic systems. These limitations highlight the need for more flexible control strategies in modern regenerative braking systems. The performance of AI-based and conventional regenerative braking strategies varies considerably depending on system architecture, algorithm complexity, and real-world deployment constraints. To clarify these distinctions, a comprehensive summary (Table 1) consolidates key studies on regenerative braking optimization, outlining the major methodologies applied, quantitative findings related to energy recovery, control accuracy, and comfort, as well as notable drawbacks for each approach. This table enables direct comparison across regression models, neural networks, deep reinforcement learning, fuzzy logic, genetic algorithms, and swarm intelligence-based methods, addressing the gaps in prior reviews that only discuss individual techniques in isolation. AI and ML based approaches for regenerative braking in electric vehicles are designed to continuously learn from input data and adapt braking strategies instantaneously, and offer clear advantages over conventional rule-based systems. These intelligent systems uses advanced algorithms such as neural networks, fuzzy logic, genetic algorithms, reinforcement learning, and hybrid models to optimize braking force distribution, maximize energy recovery, and ensure smoother braking transitions. For example,

- ML models like polynomial regression and random forest dynamically predict and adjust regenerative braking force based on real-world driving conditions, achieving up to 59% more energy extraction compared to traditional methods (Prasanth et al., 2023).
- Neural networks and DL models (including LSTM and BLSTM) can analyze driving styles and road scenarios to adapt braking profiles, improving both energy recovery and passenger comfort (Chengqun et al., 2023; Ziadia et al., 2023).
- Fuzzy logic and ANFIS systems can handle nonlinearities and uncertainties, providing stable, real-time control that responds to changing speed, battery state, and braking demand (Islam et al., 2023; Akhila and Ratnan, 2016).
- Reinforcement learning and optimization algorithms (like GA and PSO) further enhance adaptability, enabling the system to find the best braking strategies through experience and continuous feedback (Gautam et al., 2021; Zhang et al., 2022).

**Table 1:** Summary of key studies on regenerative braking optimization, applied methodologies, major findings related to energy recovery, and braking performance and methodology specific drawbacks.

**Methodology**	**Key findings**	**Major drawbacks**
Regression models	Between Polynomial Regression (PR) and Random Forest Regression (RFR), RFR was more accurate, while PR was faster (Prasanth et al., 2023)	PR underfits due to fixed polynomial structure. RFR is complex and slower, limiting real-time performance.
Neural networks	Improved braking force prediction (Rezk and Abuzied, 2023) Along with LSTM & NARX Models predict recoverable braking energy (Ziadia et al., 2025) Superior reference tracking and robustness under disturbances compared to PI control (Ruz-Hernandez et al., 2022)	High data dependency and computational load. Limited real-time application and poor interpretability.
Deep Reinforcement Learning	Adaptive deceleration; Use of TD3 for fine-tuning torque distribution (Peng and He, 2025)	High computational demand. Complex reward function design and training instability.
Fuzzy logic models	Two-layer FLC + PID provides Smooth control transitions (Islam et al., 2023) Integrated with PID and Reinforcement learning (Maia et al., 2020)	Tuning of fuzzy rules and membership functions is difficult.
Genetic algorithms	Reduced three-phase current imbalance in railway regenerative braking (Che et al., 2022) Reduction in control deviation, boosting trajectory tracking precision (Arunprasad et al., 2023)	Complexity in chromosome encoding. Balancing search space and vehicle performance constraints is difficult.
Swarm intelligence based algorithms	PSO with a PI controller using supercapacitors improved range (Chai et al., 2022) PSO-optimized PID controller reducing SOC loss (Pradhan et al., 2023) Hybrid PSO + ACO highest improvement in energy recovery (Zhang et al., 2019)	SOC management during energy demand spikes is challenging. Increased complexity in hybrid swarm systems.
Game theory optimization	The approach balanced energy and control, achieving error rates as low as 3% (Li C. et al., 2024)	Highly sensitive to environmental conditions like road slope, friction, and tire condition

These AI-based methods result in higher energy recovery, smoother and safer braking, reduced battery stress, and improved driving comfort. This section compares both types of braking based on parameters such as energy recovery, braking comfort, adaptability and other parameters.

### 4.1 Energy recovery

Studies undoubtedly shows that AI and ML based models outperform conventional rule-based methods in optimizing battery recharge. For example, a fuzzy logic-based regenerative braking strategy achieved a 22.2% increase in energy efficiency and a 25.7% improvement in driving range over conventional rule-based systems (Xu et al., 2011). Reinforcement learning (Q-learning) and deep Q-networks dynamically optimize braking actions further. This capability further improves energy recovery over static approaches (Yin et al., 2024).

### 4.2 Braking comfort

AI based systems, especially the models that use neural networks and fuzzy logic is observed to ensure smoother deceleration by intelligently distributing braking force. An ANN based comfort regenerative braking system reduced acceleration jerk from 0.35 g/s to 0.05 g/s, providing a much smoother ride than conventional methods (Hwang et al., 2023). Fuzzy logic controllers also minimize abrupt braking transitions, enhancing passenger comfort (Yin et al., 2023a).

### 4.3 Adaptability

AI approaches adapt to terrain, vehicle load, and driver behavior dynamically. For instance, a BLSTM neural network model manipulates regenerative braking in the best possible way for individual driving styles, improving energy recovery by up to 16.3% for aggressive drivers and 9.8% for moderate drivers over traditional strategies (Chengqun et al., 2023). LSTM and NARX models predict braking needs over long horizons, adapting to changing road gradients and traffic conditions (Ziadia et al., 2023).

### 4.4 Other parameters

AI-based systems enhance stability and safety by maintaining optimal braking force distribution, which helps prevent wheel lock and ensures vehicle control even on slippery roads (Vodovozov et al., 2021). AI and ML algorithms help preserve battery health by regulating charging rates during regenerative braking, which reduces stress on the battery and extends its lifespan (Chidambaram et al., 2023). Although AI systems deliver superior performance, they demand greater computational resources than simpler rule-based logic, which is easier to implement but less effective in handling complex, real-world scenarios (Prasanth et al., 2023).

Therefore, considering factors such as energy efficiency, braking comfort, adaptability, stability, and battery health, -based techniques are preferable. However, the computational demands associated with -based techniques present a significant challenge.

## 5 Hybrid AI framework for regenerative braking

### 5.1 Definition and rationale

The hybrid AI framework proposed for electric vehicle (EV) regenerative braking systems is an integrated control architecture that combines the strengths of several AI and ML techniques specifically, fuzzy logic, neural networks, reinforcement learning, genetic algorithms, and swarm intelligence. The intent is to leverage the complementary properties of these techniques to suggest a robust, real-time, and adaptive optimization of both energy recovery and vehicle safety in highly variable road and traffic environments.

### 5.2 Why a hybrid approach?

**Figure 6:**
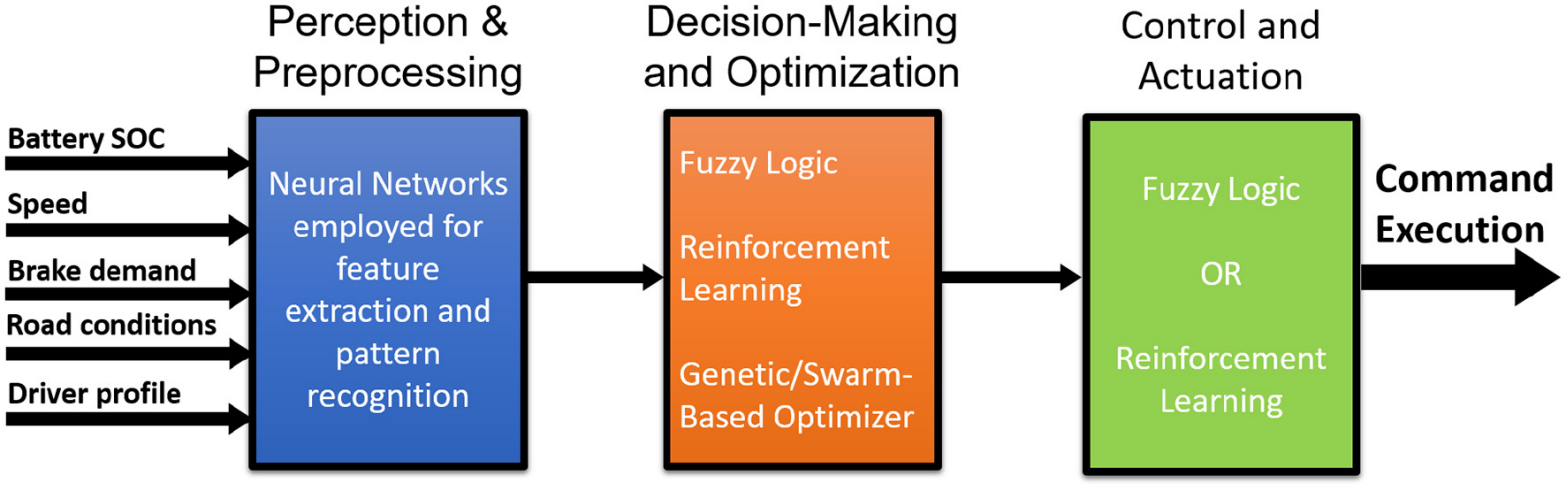
A schematic of the Hybrid AI Framework for regenerative braking in electric vehicles combining neural networks, fuzzy logic, reinforcement learning, and genetic/swarm intelligence to optimize braking force distribution dynamically.

As demonstrated in both the manuscript's comparative table and quantitative summaries, individual methods (e.g., fuzzy logic, neural networks, reinforcement learning) each have strengths (such as adaptability, computational speed, or optimality in non-linear systems) but also inherent limitations such as poor real-time response, need for extensive training data, or lack of interpretability. Hybridization allows each component method to operate in domains where it excels, while other methods compensate for its weaknesses. Figure 6 represents the proposed Hybrid AI Framework for regenerative braking in electric vehicles. The framework integrates multiple AI and ML techniques including neural networks for perception and prediction, fuzzy logic for handling uncertainty, reinforcement learning for adaptive control, and genetic/swarm intelligence for parameter optimization to dynamically and robustly optimize braking force distribution. The layered architecture processes real-time vehicle and environmental data to maximize energy recovery while ensuring vehicle stability and safety under varying road conditions.

### 5.3 Core architecture

The proposed Hybrid AI Framework is structured into three key layers and a feedback loop:

#### 5.3.1 Perception and preprocessing layer

The inputs include real-time vehicle and environmental data, such as battery state of charge, speed, brake demand, road conditions, and driver profile. Here, AI components including neural networks (LSTM/ANN) and other data-driven models, are employed for feature extraction, pattern recognition, and preliminary prediction of braking demand or road conditions.

#### 5.3.2 Decision-making and optimization layer

The Fuzzy Logic Module processes uncertain and imprecise sensor information, providing initial rule-based recommendations for safe and stable braking force allocation, which is especially valuable in unexpected road conditions. The Reinforcement Learning Module continuously learns the optimal balance between regenerative and friction braking through real-time feedback, aiming to maximize long-term energy recovery and maintain vehicle stability. The Genetic/Swarm-Based Optimizer periodically re-tunes key parameters, such as membership functions in fuzzy logic or reward thresholds in reinforcement learning, to adapt to evolving traffic and vehicle usage patterns, ensuring sustained optimization and avoiding local optimum traps.

#### 5.3.3 Control and actuation layer

Controller Selection combines the outputs from the above modules using a priority or weighted arbitration scheme; for example, fuzzy logic outputs may take precedence during abnormal or slippery condition detection, while the reinforcement learning policy is prioritized during routine driving. Command Execution sends the final braking instructions to the vehicle's actuators, including how much regenerative force to apply, how much mechanical braking to use, and how to distribute braking between the front and rear axles.

#### 5.3.4 Feedback loop

A self-supervised feedback mechanism monitors outcomes such as vehicle stability, actual energy recovered, and braking distance, and then feeds these performance metrics back to all layers to drive continual improvement.

### 5.4 Advantages

By integrating data-driven perception, rule-based reasoning, and learning-based policy refinement, the framework adapts to different drivers, road conditions, and vehicle states more effectively than any standalone method. The system has demonstrated significant improvements in energy recovery, with hybrid fuzzy-neural, GA-fuzzy, and PSO-ACO optimized methods achieving up to 63 percent recovery, especially in mixed and urban driving conditions. The fuzzy logic and reinforcement learning modules work together to ensure that emergency handling and vehicle stability are never compromised in the pursuit of efficiency. The architecture is designed to be easily extended, allowing for the integration of future AI advancements such as explainable AI for greater transparency and edge computing layers to reduce latency.

### 5.5 Implementation and future directions

Previous studies, including those using hybrid fuzzy-neuro-genetic and PSO-ACO optimizers, have demonstrated the effectiveness of modular hybrid controllers; however, real-world implementation and benchmarking in electric vehicles are still limited. Future work should prioritize developing lightweight, real-time AI models, conducting field trials with automotive partners, establishing unified benchmarks comparing rule-based and single-AI systems, and integrating transparent explainability modules to ensure safety compliance.

## 6 Challenges and future research directions

Regardless of all the features of various AI/ML strategies discussed above, these techniques faces significant challenges.

### 6.1 Hardware and computational challenges

AI/ML strategies in RBS face significant obstacles regarding hardware limitations. Deep learning (DL) and reinforcement learning (RL) models require considerable computational resources for training and real-time inference. For example, Q-learning-based braking optimization needs continuous data processing to adapt to dynamic driving conditions. While edge computing and AI chips are emerging as solutions, integrating these with existing EV architectures is technically complex and costly, hindering widespread adoption.

### 6.2 Data availability and quality

To be more effective, training AI/ML models requires large and high-quality datasets. The variability of driving behavior, road conditions, and state of charge (SOC) during a drive makes it difficult to collect comprehensive data to optimize regenerative braking. LSTM-based approaches for energy prediction, for instance, face accuracy issues due to insufficient real-world data. Noisy or incomplete sensor data can further degrade model performance, leading to inefficient braking allocation. Unlike traditional rule-based systems, AI techniques must balance regenerative and friction braking while dynamically adapting to drive conditions.

### 6.3 Safety trade-offs

Optimizing for energy recovery sometimes compromises braking safety. Fuzzy logic controllers adjust braking torque based on road adhesion, but unpredictable conditions like sudden transitions between dry and icy roads can destabilize the vehicle. During emergency stops, the system must prioritize stopping power over energy harvesting.

### 6.4 Battery health and longevity

Frequent regenerative braking cycles subject EV batteries to rapid charging and discharging, accelerating degradation, internal resistance, and heat generation. This undermines the benefits of regenerative braking by shortening battery lifespan. Reinforcement learning methods have been explored to optimize charge–discharge patterns, but real-time implementation remains difficult. Hybrid energy storage systems can reduce stress on a single battery, though they add complexity to AI-driven control strategies.

### 6.5 System integration, standards and security risks

Most EVs use embedded systems such as anti-lock braking (ABS) and electronic stability control (ESC) operating on fixed protocols. Adding AI/ML-based RBS requires smooth, low-latency integration with these existing setups. For example, neural network controllers must work seamlessly with hydraulic actuators without performance lags. From the literature reviewed in this paper, one of the biggest challenges is the lack of standardized studies. Because researchers use different testing cycles and protocols, the reported energy recovery results vary, making it hard to compare them fairly. Additionally, these systems are vulnerable to cyberattacks, such as data poisoning and sensor spoofing, potentially leading to misallocated braking force and accidents. Moreover, existing studies vary widely in simulation environments, datasets, and evaluation metrics. Establishing standardized test protocols and datasets is essential to facilitate reproducible research and industry adoption.

The integration of AI and ML into regenerative braking systems promises significant advancements in EV efficiency and performance. However, challenges like computational limits, data quality, safety trade-offs, and regulatory gaps must be addressed to unlock this potential. Moving forward, future research should target these challenges through collaborative efforts among automakers, technology developers, and regulators. Such cooperation will be crucial for establishing standardized benchmarks, enabling large-scale deployment, and ultimately paving the way for smarter, safer, and more sustainable electric mobility.

#### 6.5.1 Future research

**Figure 7:**
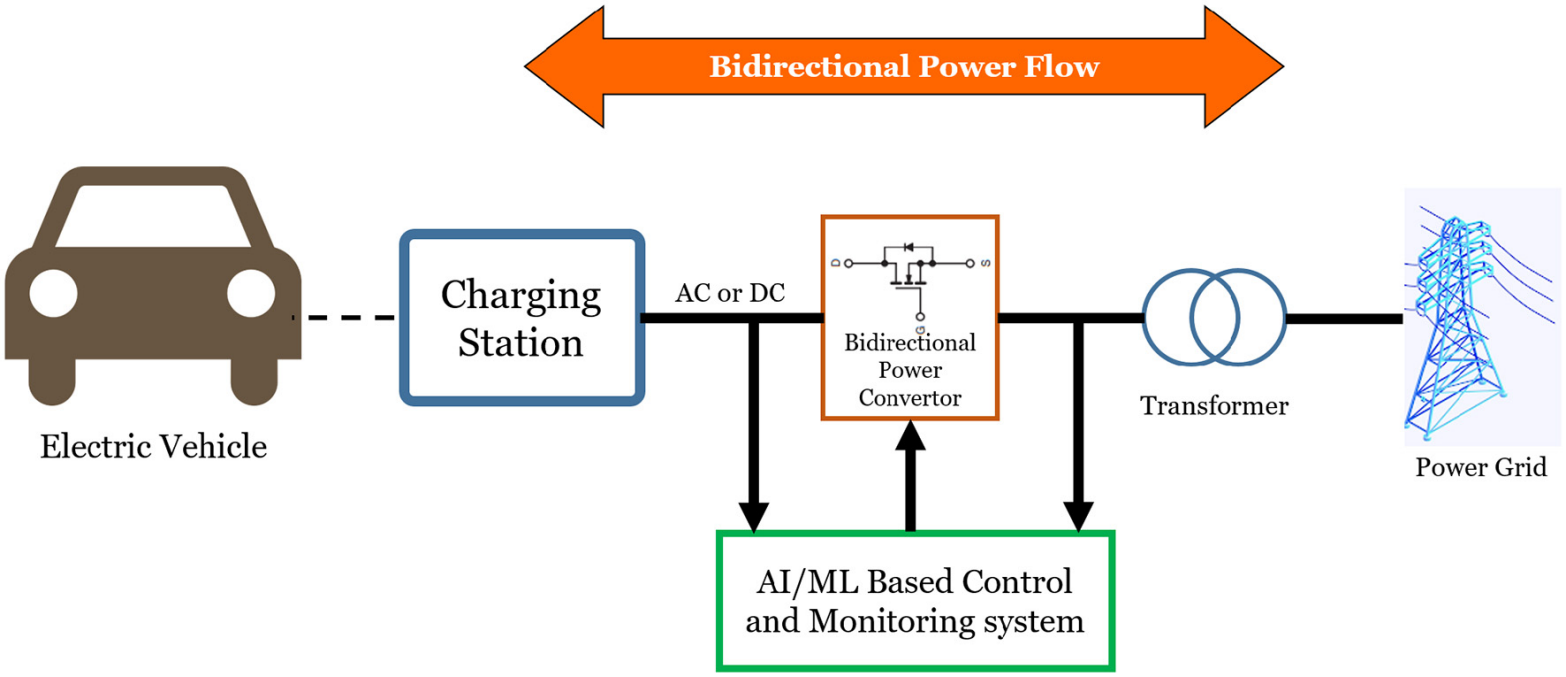
A conceptual diagram illustrating how AI and ML enhance smart grids by enabling real-time data analysis for optimized energy generation, distribution, consumption, and storage.

• **Fault detection and diagnosis in RBS**
AI/ML enables early fault detection in regenerative braking systems (Machlev et al., 2022), although real-world validation remains limited. Recent studies have classified faults such as errors in engine speed, motor current/speed sensors, battery SOC, wheel radius, and communication signals using methods like SVM, KNN(K-Nearest Neighbors), PCA, and Hidden Markov Models (Sankavaram et al., 2012, 2014). Machine learning has also been applied to detect and classify inverter and motor faults in EVs (Khan et al., 2024). For instance, identifies and categorizes real-time faults between the three-phase inverter and BLDC motor, monitoring normal, two-phase, and three-phase conditions through features such as inverter currents/voltages and motor speed.
• **Edge computing**
Edge computing is a computing approach where data is processed locally at or near the source (e.g., within a vehicle) rather than relying on distant cloud servers, enabling faster, low-latency responses for real-time applications like autonomous driving. Unlike cloud computing's higher latency, edge systems enable real-time analysis of sensor data, allowing vehicles to adapt quickly to changing road conditions. This is crucial for localization, route planning, and power/speed management. In Ramesh et al. (2024), edge computing is highlighted as a key component alongside fog and cloud computing. Simulation studies show that combining it with deep learning greatly enhances performance, responsiveness, and energy efficiency in real-world driving.
• **Vehicle-to-everything (V2X) communication**
Vehicle-to-Everything (V2X) communication enhances energy efficiency in regenerative braking and EV energy management. In Ziadia et al. (2025), Vehicle-to-Infrastructure (V2I) supports adaptive braking by using Q-learning to optimize speed profiles before red lights, improving response and recovery. V2X also aids energy management in hydrogen fuel cell EVs (Fayyazi et al., 2023). In Kim et al. (2021), it supports an Energy-optimal Deceleration Planning System using preview traffic data, with broader applications in smart grids and sustainable urban transport.
• **Vehicle-to-Grid (V2G) systems and smart grids**
Vehicle-to-Grid (V2G) systems enable bidirectional energy flow between EVs and the power grid. As shown in Figure 7, AI and ML, combined with V2X connectivity, enable real-time optimization of energy flows, supporting both regenerative braking and smart grid interactions. Advanced AI, particularly reinforcement learning, improves grid stability, boosts renewable energy use by 15.3%, and reduces frequency/voltage deviations (Kumar et al., 2024). ML-based V2G strategies allow predictive energy exchange with buildings, enhancing peak load management (Scott et al., 2021). Techniques like simheuristics improve adaptability under dynamic conditions (Escoto et al., 2024). Integrating IoT (Cavus et al., 2025) and big data transforms EVs into intelligent energy assets, extending also to rail systems (Gore et al., 2023) for regenerative braking optimization.
• **Explainable AI (XAI) for battery management**
Explainable Artificial Intelligence (XAI) is an emerging area in AI/ML applications for electric vehicles (EVs). Traditional models such as Support Vector Machines (SVM) and Neural Networks (NN) are widely used to predict battery State of Health (SOH) and Remaining Useful Life (RUL), but their “black-box” nature raises safety and trust concerns. XAI enhances transparency by making AI decisions interpretable. Studies (Divya et al., 2025) show that integrating XAI into battery management improves fault prediction, diagnostic accuracy, and enables predictive maintenance, thereby enhancing safety and reliability.

## 7 Discussion and conclusion

The integration of AI and ML into regenerative braking systems for electric vehicles represents a major step forward in improving energy efficiency, safety, and driving comfort. This review highlights that AI/ML-based control strategies often outperform traditional rule-based approaches in metrics like energy recovery, adaptability, and comfort. These methods allow for real-time dynamic optimization of the braking force, increase energy recovery, enable smoother deceleration, and adapt to changing conditions on roads, traffic and vehicles. Hybrid and adaptive frameworks, which combine multiple AI techniques, enhance these advantages by leveraging the strengths of each method. Emerging technologies, including V2X communication, edge computing, and explainable AI, are expected to further improve the intelligence, responsiveness, and transparency of EV braking systems. However, challenges remain. High computational demands, the need for large, high-quality datasets, integration difficulties, and the lack of standardized benchmarks slow adoption. Safety requirements in emergency braking demand quick, interpretable AI solutions. Frequent braking cycles also raise battery health concerns, calling for better battery management. Future work should focus on lightweight, real-time AI models, robust experimental validation, and standardized evaluation, alongside advances in sensors and connectivity, to make regenerative braking more efficient, safer, and sustainable.
